# Direct observation and measurement of circumlental space and its relation to anterior chamber angle characteristics in iridotomized phakic eyes with primary angle closure disease

**DOI:** 10.1038/s41598-024-54562-3

**Published:** 2024-02-16

**Authors:** Zhiqiao Liang, Kangyi Yang, Kun Lv, Yao Ma, Xuanzhu Chen, Zeqin Ren, Yong Liang, Xianru Hou, Huijuan Wu

**Affiliations:** Department of Ophthalmology, Peking University People’s Hospital, Eye Diseases and Optometry Institute, Beijing Key Laboratory of Diagnosis and Therapy of Retinal and Choroid Diseases, College of Optometry, Peking University Health Science Center, No. 11 Xizhimen South Street, Xicheng District, Beijing, 100044 China

**Keywords:** Circumlental space, Primary angle closure disease, Mechanisms, Partial ciliary block, Glaucoma, Anatomy

## Abstract

Primary angle closure disease (PACD) is a major cause of blindness worldwide. It has a high prevalence in East Asia, especially in China, which leads to a higher incidence of blindness than open-angle glaucoma. The aim of this study was to directly observe the circumlental space (CLS) in laser peripheral iridotomized eyes with PACD and to determine whether this structure plays a role in the pathogenesis of PACD. Fifty eyes of 50 patients with PACD, who had received laser peripheral iridotomy performed with neodymium:yttrium–aluminum-garnet were recruited from glaucoma clinics from March 2021 to May 2022, including 17 primary angle closure suspect (PACS), 16 primary angle closure (PAC) and 17 primary angle closure glaucoma (PACG). They were classified into two groups based on whether the ciliary process and the crystalline lens equator were in contact using slit-lamp photograph: the attached group and the unattached group. The demographic, clinical characteristics and anterior segment parameters measured from ultrasound biomicroscopy were compared between the attached group and the unattached group. Thirty-three eyes were assigned to the attached group and 17 eyes belonged to the unattached group. In the unattached group, the mean CLS was 0.10 ± 0.07 mm. No significant differences were identified between the different diagnosis groups in age, sex, best-corrected visual acuity, intraocular pressure, white-to-white, axial length, central corneal thickness, anterior chamber depth, flat keratometry, steep keratometry or iridotomy diameter (*p* > 0.05). The unattached group had shorter trabecular-ciliary process distance (*p* = 0.021) and larger ciliary process area (*p* = 0.001) compared with the attached group. Small CLS and its potential effect (partial ciliary block) might be considered as one of the mechanisms of PACD.

## Introduction

Primary angle closure disease (PACD) is a major cause of blindness worldwide. It has a high prevalence in East Asia,^1^ especially in China,^2^ which leads to a higher incidence of blindness than open-angle glaucoma.^3^ The mechanisms of angle closure can be described by anatomical factors that lead to aqueous flow obstruction,^1,4–6^ that have been classified as follows: 1. Pupillary block mechanism; 2. Anatomic variations at the level of ciliary body and iris (plateau iris or anteriorly placed ciliary processes); 3. Anatomic variations at the level of the crystalline lens (increased thickness or subluxation); 4. Anatomic variations posterior to the lens (increased choroidal volume or aqueous misdirection). Different mechanisms can coexist and vary by race. Primary angle closure eyes typically have certain characteristics, such as short axial length, shallow anterior chamber depth (ACD), and anteriorly placed ciliary process.^7^ However, the relationship between the crystalline lens and ciliary process has not been clearly described in the mechanism of angle closure.

In research on the progression of presbyopia, previous studies^8–11^ have focused on the circumlental space (CLS), which is the shortest distance between the ciliary process and the crystalline lens equator. Researchers have used high-resolution magnetic resonance imaging (MRI),^9,10^ goniovideography,^11^ and ultrasound biomicroscopy (UBM)^8^ to detect this area in humans and rhesus monkeys. However, these methods have their limitations in practical clinical work. MRI provides images of the whole globe and can measure the equatorial diameter of the lens, but the low spatial resolution can interfere with the accurate location of the eye and the morphometric measurement of the ciliary body. Goniovideography has been used to reflect the dynamic movement of the ciliary body during accommodation after total iridectomy in monkey eyes, but it is not feasible for human studies. UBM relies on the examined tissue structure to scatter ultrasound waves, which are then detected by ultrasound transducers. However, due to technical limitations, UBM can only effectively detect CLS in eyes with obvious crystalline lens opacity, and the test's repeatability and accuracy are relatively low due to the strict control of the orientation of the eye and UBM transducer.

Since the mid-1970s, laser peripheral iridotomy (LPI) has become the first-line treatment for PACD.^12^ LPI can promote the dynamic flow of aqueous humor in the condition of pupil block and widen the peripheral anterior chamber angle. To our knowledge, this is the first study in which researchers directly observe the relationship of the ciliary process and the crystalline lens equator through iridotomy. Our goal was to directly observe the relationship between the ciliary process and the crystalline lens in phakic eyes with PACD after LPI and to determine whether this structure plays a role in the development of PACD.

## Methods

### Patients

This case series study followed the guidelines of the Declaration of Helsinki and was approved by the Investigational Review Board and ethics committee of the Peking University People’s Hospital (Project no. 2022PHB256-001). Written informed consent was obtained from all patients.

All patients diagnosed with primary angle closure suspect (PACS), primary angle closure (PAC) and primary angle closure glaucoma (PACG) who had received LPI performed with the neodymium: yttrium–aluminum-garnet (Nd: YAG) were enrolled in this study from glaucoma clinics of University Hospital from March 2021 to May 2022. In all patients with PACD, the iridotomy was located between the 5 o'clock and the 7 o'clock, and we stopped the laser once the equatorial edge of the lens was visible and the iridotomy was functional, as evidenced by the flow of aqueous humor from the posterior chamber into the anterior chamber through the iridotomy.

PACS was defined as eyes with iris trabecular contact greater than three or more quadrants (usually functional trabecular meshwork was not visible under non-indentation gonioscopy), no peripheral anterior synechiae (PAS), and normal intraocular pressure (IOP), optic neuropathy and visual field. Normal intraocular pressure was defined as 21 mmHg or lower, and normal optic disc appearance was defined as having no signs of glaucomatous damage such as cupping, notching, thinning, hemorrhage or disc asymmetry. Normal visual field was defined as having a mean deviation and a pattern standard deviation within 95% confidence limits, and a normal glaucoma hemifield test result. An eye with iridotrabecular contact and an elevated IOP or PAS with no secondary cause for the PAS, but without glaucomatous optic neuropathy was defined as PAC. An eye with features of PAC together with glaucomatous optic neuropathy was defined as PACG.

The exclusion criteria were as follows: (1) secondary angle closure, such as iris neovascularization, iridocorneal endothelial syndrome, uveitis, lens intumescence and subluxation, and drug-induced angle closure; and (2) inability to recognize the relationship of the ciliary process and crystalline lens exactly through iridotomy; (3) the eyes had a Lens Opacity Classification System III (LOCSIII) grade over 1; (4) pseudophakic and aphakic eyes; and (5) eyes that had undergone any refractive interventions, such as add-on intraocular lens, implantable collamer lens, or corneal laser refractive surgery.

### Ophthalmologic examinations

All patients underwent a comprehensive ophthalmologic examination, including corrected visual acuity (CVA), IOP (Goldmann applanation tonometry), slit-lamp examination, and optic disc evaluation using a 90-diopter lens (Volk Optical, Inc., Mentor, OH, USA). Visual acuity was measured with the Snellen visual chart. Axial length, white-to-white (WTW), corneal diameter, central corneal thickness, ACD, flat and steep keratometry (flat K and steep K) were measured by IOLMaster biometry (IOLMaster 700, Carl Zeiss Meditec, Inc., Dublin, CA). The average values of five IOLMaster measurements with signal-to-noise ratio greater than 100 were used for analysis. Visual field perimetry (Swedish Interactive Threshold Algorithm [SITA] 24-2 test of the Humphrey visual field analyzer 750i, Carl Zeiss Meditec, Dublin, CA) was used to identify the glaucomatous visual field defects. Using an optical coherence tomography device (Heidelberg Spectralis, Heidelberg Engineering, Germany), we obtained direct visualization and measurements of retinal nerve fiber layer. Gonioscopy (Model G-4, Volk Optical, Inc., Mentor, OH) and UBM (Aviso, Quantel Medical, Inc., Bozeman, MT, USA) were performed to determine the narrow degree of the anterior chamber angle.

**Figure 1:**
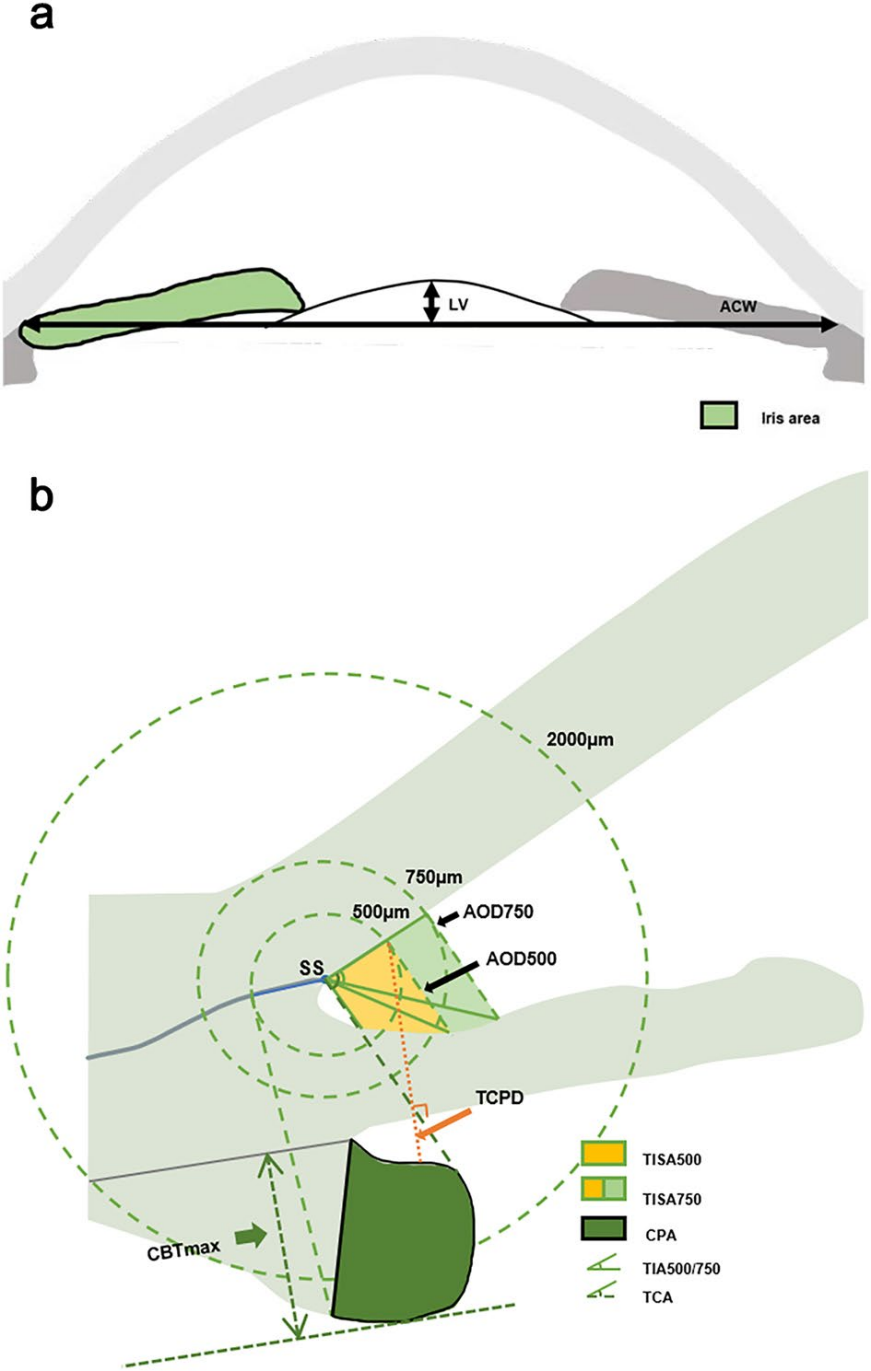
Anterior segment parameters measured by UBM. (**a**) Part of anterior segment parameters measured by UBM. (Illustrator: Kangyi Yang). LV, the perpendicular distance from the anterior pole of the lens to the horizontal line between the scleral spurs; ACW, the distance between the two scleral spurs; Iris area, the cumulative cross-sectional area of the full length (from spur to pupil) of the iris. (**b**) Part of anterior segment parameters measured by UBM. (Illustrator: Kangyi Yang). AOD 500/750: the distance between the posterior corneal surface and the anterior iris surface on a line perpendicular to the trabecular meshwork 500 μm and 750 μm from the scleral spur; TISA 500/750: the area bounded anteriorly by AOD 500 and AOD 750 as determined, posteriorly by a line drawn from the scleral spur perpendicular to the plane of the inner scleral wall to the iris, superiorly by the inner corneoscleral wall, and inferiorly by the iris surface; TIA 500/750: the apex of the angle at the iris recess and the arms of the angle passing through a point on the trabecular meshwork at 500 μm and 750 μm from the scleral spur and the point on the iris perpendicularly opposite; CPA: the cross-sectional area of ciliary process bounded laterally by a line connecting the insertion location of iris into the ciliary body and the cross-point of a line at 500 μm from the scleral spur perpendicular to the plane of the inner scleral wall to the ciliary process, and internally by the ciliary process surface; TCA: the angle between the posterior corneal surface and the anterior surface of the ciliary body; CBTMax, the thickest location of the ciliary body; TCPD, the length of the line extending from the corneal endothelium 500 μm from the SS perpendicularly through the posterior surface of iris to the ciliary process.

UBM measurements were performed using a 50-MHz transducer by a well-trained operator (Y.Y.W.) who was masked to the clinical data. Only images with a clear view of the scleral spur, angle, ciliary body, iris and anterior surface of the lens were included for analysis (Fig. 1).

Scleral spur (SS) was shown as a crucial anatomical mark in curvature of the inner surface of the angle wall, appearing as an inward extension of the sclera. The parameters we measured on full view scans at the nasal-temporal position were as follows: (1) Lens vault (LV): the perpendicular distance from the anterior pole of the lens to the horizontal line between the scleral spurs. (2) Anterior chamber width (ACW): the distance between the two scleral spurs. (3) Iris area: the area bounded by the full length (from spur to pupil) of the iris. Parameters measured on the radial scans at the superior, nasal, inferior, and temporal positions were as follows: (1) Ciliary process area (CPA): the cross-sectional area of ciliary process bounded by a line connecting the insertion location of iris into the ciliary body and the cross-point of a line at 500 μm from the scleral spur perpendicular to the plane of the inner scleral wall to the ciliary process, and internally by the ciliary process surface. (2) Maximum ciliary body thickness (CBTmax): the thickest location of the ciliary body. (3) Trabecular-ciliary process distance (TCPD): the length of the line extending from the corneal endothelium 500 μm from the SS perpendicularly through the posterior surface of iris to the ciliary process. (4) Trabecular–ciliary angle (TCA): the angle between the posterior corneal surface and the anterior surface of the ciliary body. (5) Angle-opening distance at 500 μm and 750 μm (AOD 500 and AOD 750): the distance between the posterior corneal surface and the anterior iris surface on a line perpendicular to the trabecular meshwork, 500 μm from the scleral spur. (6) Trabecular-iris angle (TIA 500 and TIA 750): the angle between the line passing through a point on the trabecular meshwork at 500 μm and 750 μm from the scleral spur, and the line from the scleral spur to the point on the iris perpendicularly opposite. (7) Trabecular–iris space area at 500 μm and at 750 μm (TISA 500 and TISA 750): the surface area bounded by AOD 500 and AOD 750 anteriorly, a line drawn from the scleral spur perpendicular to the plane of the inner scleral wall to the iris posteriorly, the inner corneoscleral wall superiorly, the iris surface inferiorly.

### Anterior segment photograph acquisition

Anterior segment photographs were taken by an experienced ophthalmologist (Z.Q.L). We used a commercially available slit lamp [SLD-701 (Light emitting diode version), Topcon Corporation, Tokyo, Japan] and a DC-4 digital camera (DC-4 Digital Camera, Topcon Corporation, Tokyo, Japan). The digital camera was mounted on the clinic slit lamp, converting it into an ophthalmic imaging instrument. DC-4 can be used with EZ Capture Software (Topcon Corporation, Tokyo, Japan) and IMAGEnet® 4 Digital Imaging Systems (Topcon Corporation, Tokyo, Japan) for clear and sharp imaging of the anterior segment of the eye. The experienced ophthalmologist (Z.Q.L) magnified the region of interest (iridotomy zone in our study) to required magnification, and set the camera on an autofocus function to focus the image. In addition to standard slit lamp illumination, neither system used additional illumination. For the purpose of measurement in this study, we standardized the magnification and settings as much as possible. An experienced ophthalmologist (Z.Q.L) took 2 good-quality diffuse illumination images and 2 good-quality direct focal illumination images for at least one patient. The definitions of diffuse illumination and direct focal illumination were as follows. 1. Diffuse illumination: To obtain the largest field of view, a wide beam of light was directed at the anterior segment of the eye at low magnification using a diffuse filter. 2. Direct focal illumination: A slit beam of light was obliquely focused on the site of iridotomy. The angle between the microscope and illumination system was 45 degrees. One or two ciliary processes could be seen, and in two processes eyes, the larger one was selected to be studied. All eyes were divided into two groups based on whether the ciliary process and the crystalline lens equator were attached or not. The photographs were taken under natural conditions, without mydriasis or miosis, one week after laser iridotomy. Eyes categorized in the attached group were defined as at least one ciliary process and the crystalline lens equators were attached to each other.

### Imaging analysis

**Figure 2:**
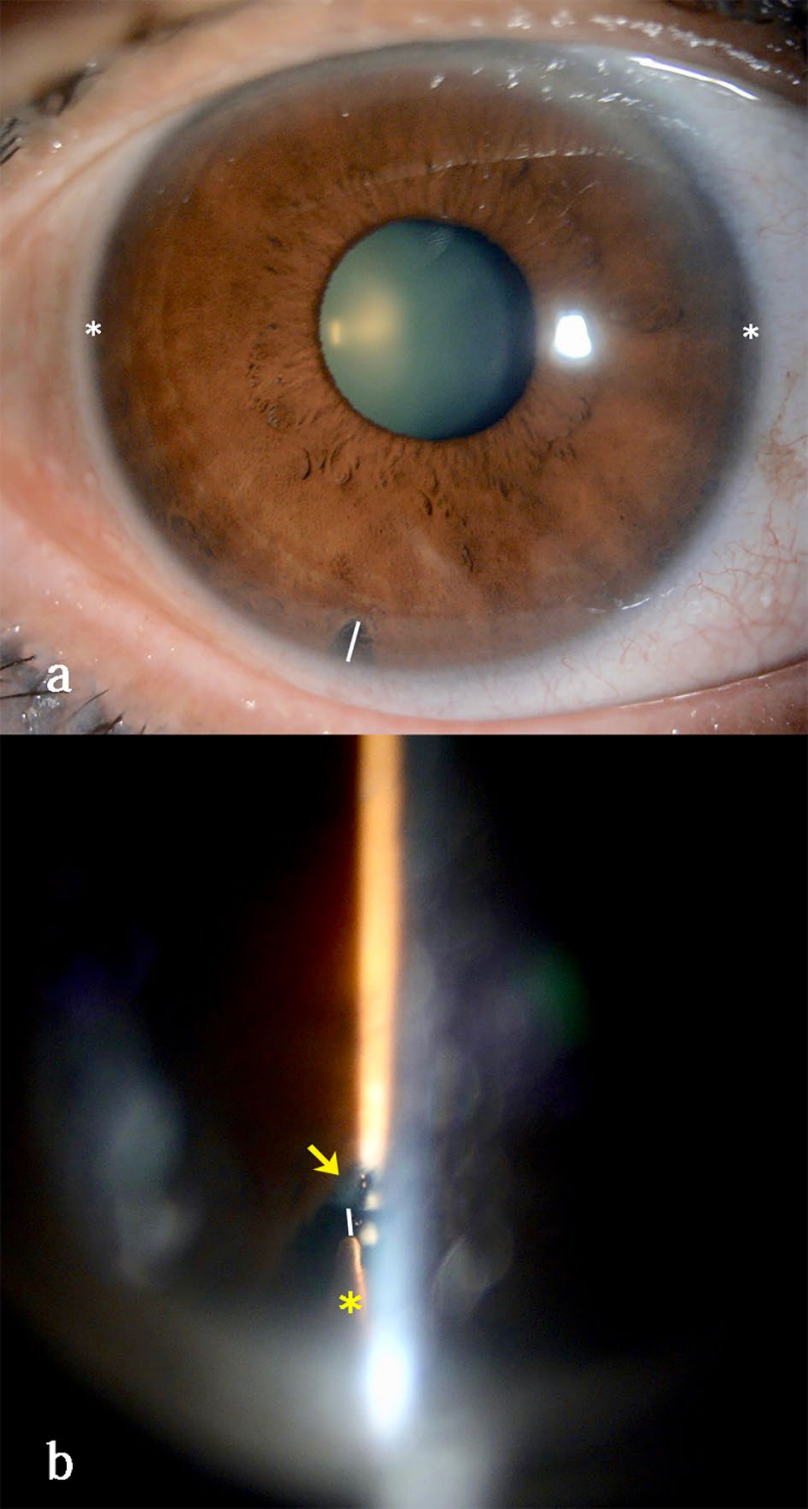
Schematic diagram for calculating circumlental spaces. (**a**) Diffuse illumination photograph of a 60-year-old female with primary angle closure suspect. Utilizing ImageJ software, the diameter of the iridotomy (white line) could be calculated according to the white-to-white (the distance between two white stars) measured by IOLMaster700. (**b**) Direct focal illumination photograph of the same eye. The circumlental space (white line) could be calculated using ImageJ software based on the known diameter of the iridotomy. The yellow arrow and star represent the crystalline lens and ciliary process, respectively.

We used ImageJ software (Java-based image-processing and analysis software 1.8.0_172, National Institutes of Health, USA) to calculate the radial diameter of iridotomy in both groups and CLS in every image in the unattached group (Fig. 2a,b) according to the anterior segment parameters measured by IOLMaster 700. CLS was defined as the shortest distance between the ciliary process and crystalline lens equator. In the diffuse illumination image, the radial diameter of each iridotomy could be calculated according to the WTW measured by IOLMaster 700. In the direct focal illumination image, the CLS could be calculated based on the known radial diameter of each iridotomy. The specific calculation methods were as follows. First, in the diffuse illumination image, we selected the line segment between the 3 and 9 o’clock horizontal limbuses. In the set scale window, we displayed the length of the line in pixels. Then, we typed the known WTW and the unit of measurement in the appropriate boxes and clicked OK. After that, we selected the line segment of the iridotomy and obtained the actual length of the iridotomy (Fig. 2a). We utilized a similar method to calculate the CLS in the direct focal illumination image (Fig. 2b).

### Repeatability and reproducibility

We performed repeatability and reproducibility analysis of CLS and UBM parameters. Ten patients in each group were randomly selected for analysis. The first observer (K.Y.Y) measured CLS twice within two weeks to test intra-observer variability. A second observer (Z.Q.L) measured the same images independently on a different day to decide inter-observer variability. The intra-observer and inter-observer variability were calculated using the coefficient of the intra-class correlation (ICC).

### Statistical analysis

The measurements were analyzed using software SPSS 24.0 (SPSS, Inc., Chicago, IL, USA). The mean and standard deviations were calculated for continuous data (age, CVA, IOP, WTW, axial length, CCT, ACD, flat K, steep K, diameter of iridotomy, CLS, TCPD, TCA, CPA, CBTMax, ACW, LV, TIA500/750, AOD500/750, TISA500/750, iris area). The normality of the data was tested by the Kolmogorov–Smirnov test. The continuous data were analyzed by one-way analysis of variance followed by multiple comparisons using Dunnett’s T3 test. Binary variables (sex and different diagnosis) were tested by Pearson’s chi-square test / Fisher exact test. A P value of less than 0.05 was considered to be statistically significant.

## Results

Fifty eyes of 50 patients, 17 PACS eyes, 16 PAC eyes, and 17 PACG eyes were enrolled in this study. Thirty-three eyes (66%) were assigned to the attached group, and 17 eyes (34%) belong to the unattached group. In the attached group, the number of PACS, PAC and PACG were 9, 12, and 12 respectively. In the unattached group, the number of PACS, PAC and PACG were 8, 4, and 5 respectively. The attached group and the unattached group did not show significant differences in diagnosis (*p* = 0.363).

The baseline characteristics of the patients are listed in Table 1. The mean age of the patients was 62.5 ± 8.1 years. Thirteen patients were male and 37 were female. The mean CVA was 0.71 ± 0.26. No significant differences were identified between the different diagnosis groups in age, sex, CVA, IOP, WTW, axial length, CCT, ACD, flat K, steep K or radial diameter of iridotomy (*p* > 0.05) (Table 1).

**Table 1:** Baseline characteristics of the enrolled patients in three groups.

Variables		PACS	PAC	PACG	*p*
Cases (eyes)	50	17	16	17	
Gender (male/female)	13/37	2/15	4/12	7/10	0.194
Age (years)	62.5 ± 8.1	62.2 ± 9.6	62.4 ± 6.4	63.1 ± 8.1	0.951
CVA (Decimal)	0.71 ± 0.26	0.80 ± 0.28	0.66 ± 0.21	0.67 ± 0.27	0.256
IOP (mmHg)	16.1 ± 4.6	15.0 ± 2.5	15.7 ± 3.8	17.3 ± 6.4	0.367
White-to-White (mm)	11.5 ± 0.39	11.5 ± 0.42	11.4 ± 0.35	11.6 ± 0.40	0.663
Axial length (mm)	22.3 ± 0.8	22.1 ± 0.7	22.2 ± 1.1	22.6 ± 0.6	0.236
CCT (um)	547.9 ± 40.1	544.0 ± 44.9	555.9 ± 40.1	544.9 ± 36.6	0.698
ACD (mm)	2.4 ± 0.3	2.4 ± 0.4	2.4 ± 0.3	2.4 ± 0.3	0.688
Flat K (Diopter)	44.4 ± 1.5	44.7 ± 1.2	44.4 ± 1.9	43.9 ± 1.5	0.343
Steep K (Diopter)	45.7 ± 3.5	45.3 ± 1.1	46.1 ± 1.6	45.6 ± 3.5	0.698
Diameter of iridotomy (mm)	0.80 ± 0.25	0.88 ± 0.31	0.77 ± 0.17	0.68 ± 0.13	0.195

*PACS* primary angle closure suspect; *PAC* primary angle closure; *PACG* primary angle closure glaucoma; *CVA* corrected visual acuity; *IOP* intraocular pressure; *CCT* central corneal thickness; *ACD* anterior chamber depth; *Flat K* flat keratometry; *Steep K* steep keratometry.

**Figure 3:**
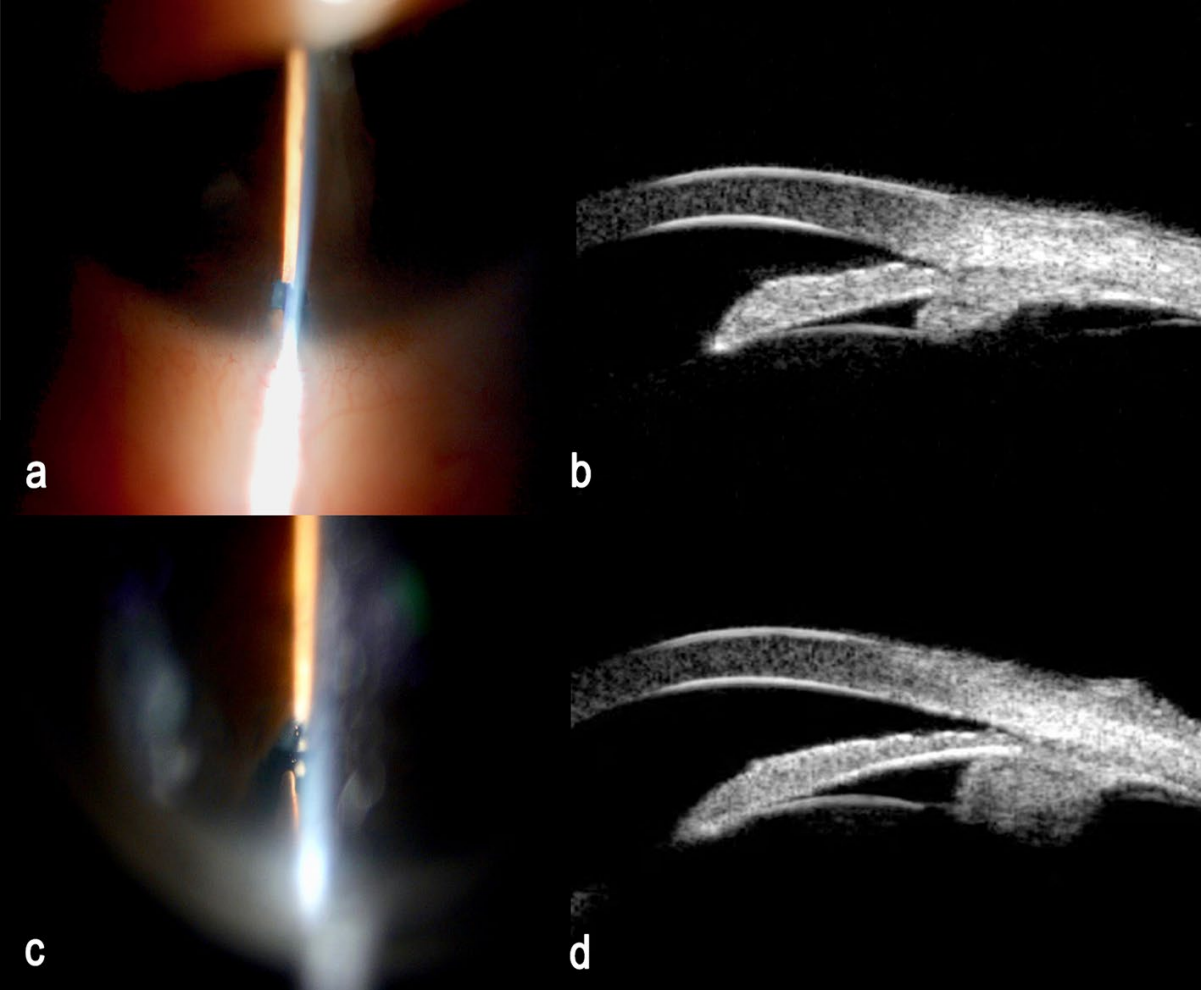
Representative cases of the attached group and the unattached group in this study. (**a**) An anterior segment photograph of a 66-year-old female with a diagnosis of chronic primary angle closure glaucoma. The ciliary process and the crystalline lens equator were attached. (**b**) UBM of the same patient showed that the ciliary body was small and posterior located. (**c**) An anterior segment photograph of a 67-year-old female with a diagnosis of primary angle closure suspect. The ciliary process and the crystalline lens equator were unattached. The circumlental space was 0.094 mm. (**d**) UBM of the same patient showed that the ciliary body was large and anteriorly rotated.

The comparison of demographic, clinical characteristics and anterior segment UBM parameters between the attached group and the unattached group are shown in Table 2. No significant differences were identified in age, diagnosis, CVA, IOP, WTW, axial length or ACD (*p* > 0.05) (Table 2). The proportion of men in the attached group was significantly higher than in the unattached group (*p* = 0.038). The unattached group had a shorter TCPD (*p* = 0.021) and a larger CPA (*p* = 0.001) than the attached group (Fig. 3). In the unattached group, the mean CLS was 0.10 ± 0.07 mm.

**Table 2:** Comparison between attached and not attached groups.

Variables		Attached group	Not attached group	*p*
Cases (eyes)	50	33	17	
Diagnosis PACS/PAC/PACG	17/16/17	9/12/12	8/4/5	0.363
Demographic and clinical characteristics				
Gender (male/female)	13/37	12/21	1/16	0.038
Age (years)	62.5 ± 8.1	61.7 ± 8.9	64.2 ± 6.5	0.323
CVA (Decimal)	0.71 ± 0.26	0.73 ± 0.27	0.67 ± 0.25	0.455
IOP (mmHg)	16.1 ± 4.66	16.3 ± 5.2	15.6 ± 3.1	0.663
White-to-White (mm)	11.5 ± 0.4	11.5 ± 0.4	11.5 ± 0.4	0.750
Axial length (mm)	22.3 ± 0.8	22.2 ± 0.8	22.5 ± 0.8	0.293
ACD (mm)	2.4 ± 0.3	2.3 ± 0.2	2.5 ± 0.4	0.184
Diameter of iridotomy (mm)	0.8 ± 0.2	0.78 ± 0.19	0.83 ± 0.31	0.595
CLS (mm)	–	0	0.10 ± 0.07	–
Parameters on UBM				
TCPD	0.54 ± 0.96	0.56 ± 0.10	0.49 ± 0.07	0.021
TCA	53.7 ± 9.0	53.4 ± 9.6	51.1 ± 7.6	0.456
CPA	0.58 ± 0.14	0.54 ± 0.12	0.68 ± 0.12	0.001
CBTMax	1.2 ± 0.1	1.2 ± 0.1	1.2 ± 0.1	0.678
ACW	11.3 ± 0.4	11.2 ± 0.4	11.3 ± 0.5	0.881
LV	0.89 ± 0.26	0.87 ± 0.23	0.93 ± 0.31	0.505
TIA500	10.4 ± 6.5	10.8 ± 7.1	9.5 ± 5.2	0.594
TIA750	9.8 ± 6.4	9.8 ± 6.9	9.7 ± 5.5	0.962
AOD500	0.10 ± 0.07	0.10 ± 0.07	0.09 ± 0.05	0.521
AOD750	0.13 ± 0.09	0.13 ± 0.09	0.13 ± 0.08	0.873
TISA500	0.03 ± 0.02	0.03 ± 0.02	0.03 ± 0.02	0.746
TISA750	0.05 ± 0.04	0.06 ± 0.04	0.06 ± 0.03	0.836
Iris area	2.0 ± 0.2	1.98 ± 0.21	2.00 ± 0.20	0.778

*PACS* primary angle closure suspect; *PAC* primary angle closure; *PACG* primary angle closure glaucoma; *CVA* corrected visual acuity; *IOP* intraocular pressure; *ACD* anterior chamber depth; *CLS* circumlental space; *TCPD* trabecular-ciliary process distance; *TCA* trabecular–ciliary angle; *CPA* ciliary process area; *CBTmax* the thickest location of the ciliary body; *ACW* anterior chamber width; *LV* lens vault; *AOD 500/750* angle opening distance 500/750 μm from the scleral spur; *TIA 500/750* trabecular-iris angle at 500/750 μm; *TISA 500/750* trabecular-iris space area at 500/750 μm. UBM parameters measured in this study.

The intra-observer ICC ranged from 0.840 to 0.979, and the inter-observer ICC ranged from 0.844 to 0.971 (Table 3), which showed good repeatability and reproducibility of all parameters.

**Table 3:** Intra-observer and Inter-observer intra-class coefficients of parameters.

Parameters	Intra-class coefficients	
	Intra-observer	Inter-observer
Diameter of iridotomy	0.904	0.887
CLS	0.901	0.890
TCPD	0.922	0.877
TCA	0.947	0.880
CPA	0.883	0.856
CBTMax	0.903	0.893
ACW	0.881	0.897
LV	0.853	0.849
TIA500	0.904	0.907
TIA750	0.952	0.912
AOD500	0.891	0.886
AOD750	0.951	0.844
TISA500	0.979	0.936
TISA750	0.840	0.849
Iris area	0.921	0.971

*CLS* circumlental space; *TCPD* trabecular-ciliary process distance; *TCA* trabecular–ciliary angle; *CPA* ciliary process area; *CBTmax* the thickest location of the ciliary body; *ACW* anterior chamber width; *LV* lens vault; *AOD 500/750* angle opening distance 500/750 μm from the scleral spur; *TIA 500/750* trabecular-iris angle at 500/750 μm; *TISA 500/750* trabecular-iris space area at 500/750 μm.

## Discussion

This study reported the relationship between the ciliary process and crystalline lens of 50 phakic eyes with YAG laser iridotomy, of which 33 eyes (66%) were attached and 17 eyes (34%) were not. The mean CLS was 0.10 ± 0.07 mm in the unattached group, to the best of our knowledge, no other studies have previously explored the role of CLS in PACD in humans. This novel discovery may provide new evidence for further understanding the pathogenesis of PACD.

Few studies have focused on CLS and have mostly explored the role of CLS in presbyopia. Previous studies^11,13,14^ have found a significant decrease in CLS with age in monkey eyes, a characteristic of the aging human eye that has been documented previously in vitro with scanning electron microscopy^15^ and in vivo with MRI^9,10,16,17^ and UBM^8^. Kasthurirangan et al. proposed that in the 40-year-old age gap between young and old people, the CLS is reduced by 40%.^17^ Strenk et al. suggested that both nasal and temporal CLS decreased by approximately 0.47 mm by the age of 80.^9^ As previously presented,^9,10,16^ the decrease in CLS was mainly due to the decrease in ciliary muscle ring diameter, and the length of the lens equator was constant with age. There were a few possible explanations for the above aging changes.^16^ First, this might be secondary to the increase in lens thickness. There was evidence that due to the increase in anterior and posterior lens cortices, inward tension was applied to the ciliary muscle without altering the lens diameter. Because there are a large number of zonulas attached to the anterior surface of the lens, the axial growth of the lens with age might exert an inward pull to the ciliary process, resulting in a reduction in CLS. On the other hand, as previously proposed by Bito and Miranda,^18^ the primary cause of the decrease in the diameter of the ciliary ring might be the increase in the resting length of restoring elasticity (choroid). In this case, the inward movement of ciliary muscle with age would reduce the tension on the lens and would lead to an increase in the thickness of the lens, and lens thickening may be the result of a decrease in ciliary ring diameter. In addition, the decrease in ciliary ring diameter might also be due to the growth and rearrangement of ciliary muscle itself.

Croft et al. reported that the CLS of young and middle-aged people was 0.66 ± 0.03 mm (grouped together) and that of elderly people was 0.35 ± 0.05 mm in a UBM study based on healthy human subjects.^8^ A key finding of the present study was that among 50 iridotomized phakic eyes with PACD, 33 eyes showed the crystalline lens and the ciliary process were attached to each other under direct view. In the unattached group, the mean CLS was 0.10 ± 0.07 mm, which was much shorter than that in the previous study. This interesting discovery may indicate that a small CLS may be one of the critical factors for the development of PACD.

Anterior positioning of ciliary processes was thought to be a predisposing factor for PACD,^19^ and it was present in plateau iris configuration eyes.^20^ Previous studies have proven that Chinese individuals have a more anteriorly positioned lens and more anteriorly positioned ciliary processes than Caucasians.^21,22^ In the current study, 66% of the PACD eyes showed no space between the ciliary process and crystalline lens, and the percentage was even higher in PAC (75%) and PACG (70.6%) eyes. The other 34% of the cases showed a significantly smaller CLS compared with previous data. Theoretically, these structural characteristics may affect aqueous humor (secreted from the posterior ciliary process) moving forward at the ciliary-lens level by increasing flow resistance. These changes in flowability might result in partial ciliary block; that is, part of the aqueous humor accumulates behind the crystalline lens, forming a vicious circle,^1,4^ and then when there is a significant posterior-to-anterior pressure difference, the lens and iris move forward, leading to gradual angle closure and increased pressure in front of the lens and iris. When the pressure difference tends to balance because of posterior pulling from lens zonule and increased pressure in front of lens, the forward movement of the lens and iris stops. In our previous case reports,^23^ we described two cases of PACG patients who had persistent shallow anterior chamber after phacoemulsification, intraocular lens implantation and goniosynechialysis. The anterior chamber depth gradually increased after surgical iridozonulohyaloidovitrectomy or YAG laser iridozonulohyaloidotomy, suggesting that partial ciliary block may play a role in the pathogenesis of PACG. Partial ciliary block is different from classical ciliary block glaucoma. The latter one is a complete block, and the huge pressure difference makes the lens and iris completely move forward and attach to the cornea. In partial ciliary block, the forward force is not as large as in the condition of complete block. Therefore, various degrees of pressure difference make the lens and iris move forward in varying degrees, which might potentially explain why patients with PACD show different degrees of shallow anterior chamber and lens vault clinically. Schwartz et al. presented a case demonstrating an acute angle-closure glaucoma, instigated by the build-up of retrovitreal fluid. This accumulation triggers an anterior shift of the vitreous face and the lens-iris diaphragm, remarkably without any preceding surgical intervention or miotics.^24^ This mechanism may affect some patients with PACD and may also occur in certain stages of the disease.

Another finding of present study was that patients in the unattached group had a larger CPA and a shorter TCPD than those in the attached group. We suspect that this may be due to the fact that in patients with a relative larger CLS in unattached group, sufficient space allows the ciliary process tissue to stretch and relax, resulting in a larger cross-sectional area. At the same time, sufficient space also allows the ciliary process to rotate more anteriorly under the pressure gradient.

Our study also had some limitations. There was no controlled group in this study because of ethical reasons, so we could not compare the CLS between patients with PACD and healthy elderly individuals. Although this issue may reduce the strength of our results, the mean CLS value in the unattached group (0.10 mm) measured in this study was much smaller than those reported in the literature (0.35 mm).^8^ On the other hand, we enrolled a relatively small number of cases. Despite this, we found that the ciliary process and crystalline lens were attached in 66% of eyes with PACD.

This study has demonstrated that the CLS in PACD eyes was small or even near zero, and partial ciliary block resulting from small CLS might be considered as one of the potential mechanisms of PACD.

## Data Availability

Datasets from the current study are not publicly available due to compliance to privacy. Summary statistics are available from the corresponding author on reasonable request.
